# Fouling-free ultrafiltration for humic acid removal†

**DOI:** 10.1039/c8ra03810d

**Published:** 2018-07-11

**Authors:** Hassan Younas, Jiahui Shao, Yiliang He, Gul Fatima, Syed Taseer Abbas Jaffar, Zohaib Ur Rehman Afridi

**Affiliations:** School of Environmental Science and Engineering, Shanghai Jiao Tong University Shanghai 200240 China jhshao@sjtu.edu.cn +86-21-54742127; The State Key Laboratory of Materials Oriented Separations, College of Chemical Engineering, Nanjing Tech University Nanjing 210009 China; Department of Chemistry and Chemical Engineering, Syed Babar Ali School of Science and Engineering, Lahore University of Management and Sciences Lahore 54000 Pakistan; Department of Civil Engineering, Shanghai Jiao Tong University Shanghai 200240 China; Energy Management and Sustainability, U.S. Pakistan Centre For Advanced Studies in Energy, UET Peshawar Pakistan

## Abstract

Membrane fouling is a serious concern that significantly affects the membrane filtration process. In this study, an ultrafiltration (UF) membrane was developed with surface auto-regeneration potential by immobilizing a photocatalyst [titanium dioxide nanoparticles (TiO_2_ NPs)] on a hybrid polyvinylidene fluoride (PVDF) membrane to reduce fouling. The combination of photocatalysis and UF, namely, photocatalytic UF, induced the surface auto-regeneration potential to the membrane. The photocatalytic process was initiated after UV light reached the TiO_2_ NPs through a quartz window in the membrane containing cell. The membrane, with an optimized distribution of TiO_2_ NPs (3.04 g m^−2^), could completely regenerate itself during photocatalytic UF [with 2 mg L^−1^ humic acid (HA)] without experiencing membrane fouling during 90 min of filtration. The impact of temperature, an important factor for increasing the kinetic rate of the photocatalyst, was also studied. The results showed that an increase in temperature did not affect the photocatalytic process, but increased the permeate flux, which was attributed to the decrease in kinematic viscosity of the water. Finally, four consecutive photocatalytic UF cycles demonstrated the stability of the membrane for a fouling-free UF process.

## Abstract 2

Membrane fouling is a serious concern that significantly affects the membrane filtration process.

## Graphical abstract

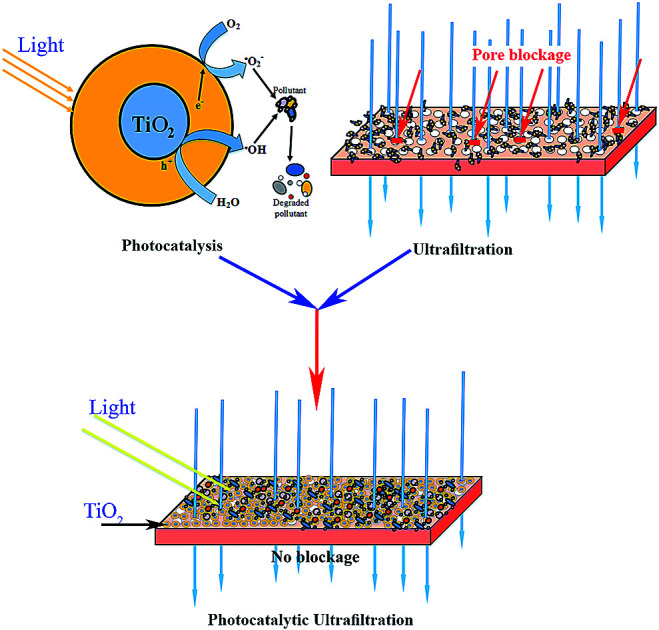

## Introduction

Membrane technology has emerged as an efficient technology for physical separation of pollutants. Polymer membrane filtration has been an effective water treatment solution for decades, and UF is a well-established filtration process to treat a broad range of contaminated water sources due to its low energy requirement, easy automation, and optimal quality of treated water.^1^ Despite these advantages, organic fouling is a severe challenge that restricts the broad and frequent applications of membrane technology.^2^ Due to the high demand of sustainable solutions for fouling mitigation, several membrane modification procedures have been introduced that reduce fouling at significant levels,^3,4^ and new modification procedures are being developed to increase the membrane filtration efficiency.

PVDF is a widely used polymer for membrane fabrication due to its strong resistance to chemical and physical degradation, biological oxidation, and irradiation deterioration.^1,5,6^ However, PVDF is hydrophobic,^7,8^ which increases the susceptibility of PVDF-based membranes for fouling.^9^ Therefore, PVDF membranes are modified with hydrophilic additives including hydrophilic polymers,^10,11^ amphiphilic copolymers,^12,13^ and inorganic nanoparticles,^10,14–16^ to reduce the hydrophobicity.^1,17^ The modification of the PVDF membrane by different types of additives increases its hydrophilicity, resulting in a decrease in fouling.^18–20^

In recent years, the application of NPs has played a prominent role in membrane filtration, where NPs are applied as an integral part of the membrane.^17,21,22^ The use of NPs alters the basic characteristics of the membrane, among which hydrophilicity remains the most important factor that reduces membrane fouling.^23–28^ The NPs attract more water molecules through hydrogen bonding and produce a thin layer of water over the membrane surface.^29^ As a result, the adsorption of foulant on the membrane is reduced and fouling of the membrane is avoided to a large extent.

Among several types of NPs, TiO_2_ NPs are highly stable and well established at commercial levels. TiO_2_ NPs are widely renowned for their photocatalytic oxidation potential.^30,31^ TiO_2_ NPs generate highly energetic electron–hole pairs when energy-rich photons (energy greater than the band gap of TiO_2_) strike the TiO_2_ surface. The electron jumps from the valance band to the conduction band and leave an empty place, which is known as a hole. Thus, the hole is an arbitrary positively charged species that reacts with water to produce a reactive oxygen species (ROS), ˙OH. The electron reacts with molecular oxygen and generates another ROS, namely, the superoxide radical anion, ˙O^−2^. Both ROS species possess great potential to oxidize a wide range of pollutants.^32,33^ The commercially available P25 TiO_2_ NPs are applied in different environmentally-based research studies to induce photocatalytic degradation of contaminants,^31^ which include dye molecules,^34,35^ organic pollutants,^30,36^ and microorganisms.^37,38^

TiO_2_ NPs are also applied in polymer membranes. The application of TiO_2_ NPs in the membrane has been shown to improve the antifouling characteristics of the membrane.^39^ Ngang *et al.*^40^ applied P25 TiO_2_ NPs in the PVDF membrane and found less membrane fouling during UF of methylene blue. Madaeni *et al.*^41^ prepared a cellulose/TiO_2_ hybrid membrane and the resultant membrane was resistant to fouling. The improvement in antifouling characteristics is linked with the increase in hydrophilicity of the membrane.^24^ Also, NPs-containing membranes show self-cleaning capacity when TiO_2_ NPs are activated under UV light after fouling.^10^ However, the activation of TiO_2_ NPs during filtration has seldom been reported. In a recent study, Fischer *et al.*^42^ deposited TiO_2_ nanotubes on a polyether sulfone (PES) microfiltration membrane *via* an anodization method. They only reported the photocatalytic degradation of diclofenac by immobilized TiO_2_ nanotubes and did not report any antifouling potential of the as-prepared membranes. For instance, it was hypothesized that UV activation of surface immobilized TiO_2_ NPs would eliminate fouling of the membrane during UF, resulting in a fouling-free UF process.

In this study, the PVDF-based membrane was developed with surface located TiO_2_ NPs and UF of HA was conducted while activating TiO_2_ NPs during the filtration process. The membrane surface was irradiated with UV light to achieve a fouling-free UF process by activation of TiO_2_ NPs. The distribution of TiO_2_ NPs on the membrane surface was optimized using contact angle, membrane internal resistance, and filtration results. The impact of HA concentration was also studied to estimate the suitable concentration of pollutant for a sustaining fouling-free UF process. Finally, the stability of the membrane for fouling-free UF was examined through four consecutive cycles.

## Materials and methods

### Materials

PVDF (SOLEF® 6020) was purchased from Solvay Ltd. TiO_2_ NPs (P25) were purchased from Degussa Corp and HA was purchased from Aldrich. All other chemicals used in this study were analytical grade and purchased from Sinopharm Chemical Reagent Corp. (SCRC), China, unless otherwise stated.

### Membrane preparation

In our previous study, we optimized the membrane constituents, namely PVDF, PEG, and TiO_2_ NPs, inside the membrane matrix.^43^ In this study, TiO_2_ NPs were immobilized on the surface of the hybrid membrane with the optimized membrane components, PVDF (12 wt%), PEG (2 wt%), and TiO_2_ NPs (1.5 wt%), based on the previous study.

The membranes were fabricated using a conventional yet significant method, *i.e.*, the phase inversion method.^10^ In addition to the phase inversion method, a procedure was introduced to immobilize TiO_2_ NPs on the membrane surface. In detail, the casting solution was prepared by adding polymer (PVDF), pore forming agent (PEG), and hydrophilic additive (TiO_2_ NPs) in dimethylacetamide (DMAc). The mixture was mechanically stirred at 250 rpm and 40 °C for 24 h. Then, the mixture was left undisturbed overnight at 40 °C (without stirring) for degassing. After complete degassing, a glass plate was immobilized with TiO_2_ NPs; the required amount of TiO_2_ NPs was sonicated in 10 mL ethanol. A well-dispersed suspension of TiO_2_ NPs was poured gently on the middle of a clean glass plate. After the suspension covered the designated area, the glass plate was left to air dry. Then, the TiO_2_ NPs-containing glass plate was kept in a dry heat oven at 50 °C for 5 min to ensure complete evaporation of ethanol, followed by the casting of the polymer solution on a glass plate with a Doctor's blade (the blade height and speed were adjusted to 200 μm and 1.2 m min^−1^, respectively). After spreading of the casting solution, the glass plate was transferred into a membrane coagulation bath at room temperature. After complete coagulation of the membrane solution, the membrane was transferred into ultrapure water and kept overnight to achieve complete removal of the solvent from the membrane.

### Membrane characterization and analytical methods

The as-prepared membranes were characterized for their hydrophilicity and resistance. The hydrophilicity of the membrane was assessed by contact angle measurements using a goniometer (MAIST Vision). The surface dried membrane was fixed on a glass slide, and a 5 μL water droplet was dropped onto the membrane. The contact angle was measured after 0.03 s of contact between the membrane and the droplet.

The membrane internal resistance was calculated by using the membrane for pure water flux. The membrane was pre-compacted by filtering DI water for 30 min using a cross-flow filtration system, and the volume of permeate water was collected after pre-compaction. The internal resistance of the membrane was calculated using eqn (1):1
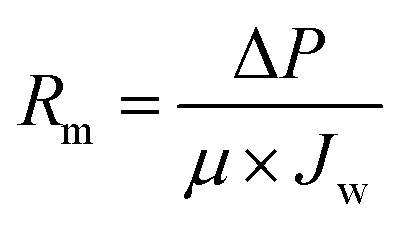
where *R*_m_ is the internal resistance of the membrane, Δ*P* is the applied pressure (0.1 MPa), *μ* is the viscosity of pure water, and *J*_w_ is the pure water flux.

### Photocatalytic UF

**Fig. 1:**
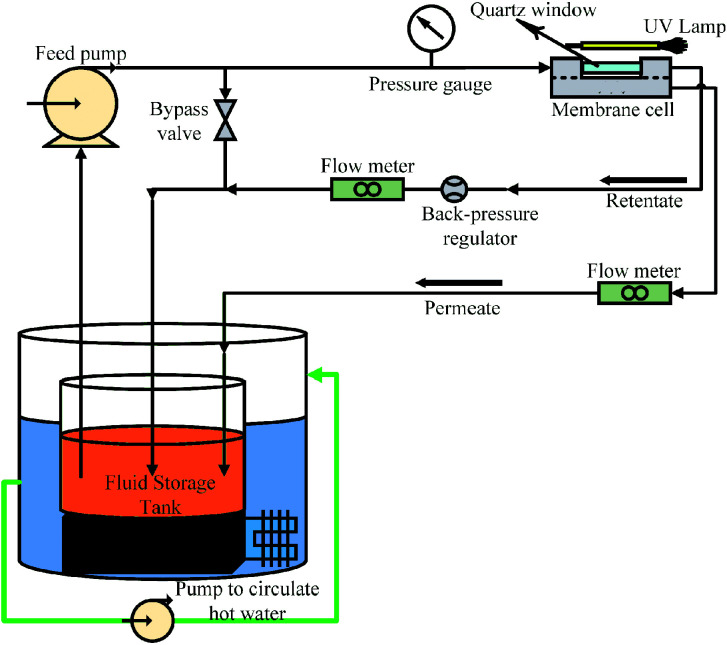
A schematic of photocatalytic UF system (operational pressure: 0.1 MPa, cross-flow rate: 0.5 L min^−1^).

The photocatalytic UF experiments were conducted in a laboratory scale custom-made cross-flow filtration unit. The filtration system consisted of a fluid storage tank with a thermo-regulator to control temperature, a pump to circulate water in the membrane cell, flow meters to control the flow over the membrane, a pressure regulator to adjust pressure, and a membrane-containing cell. The membrane cell contained a membrane with an active area of 48 cm^2^ (8 cm × 6 cm), which was illuminated with UV light through a quartz window on top of the membrane cell. A detailed schematic of the photocatalytic UF system is shown in Fig. 1.

The cross-flow rate of feed was kept constant at 0.5 L min^−1^ and the pressure was maintained at 0.1 MPa. A high-pressure mercury lamp provided irradiation of UV light with the maximum light emitting capacity at 365 nm and a light intensity of 1.2 mW cm^−2^.

Before the filtration experiment, each membrane was filtered with approximately 100 L m^−2^ ultrapure water to attain a stable water flux and constant compaction. The photocatalytic UF was carried out for a duration of 90 min. The samples from the permeate as well as the feed were collected at a regular intervals of 10 min. The concentration of HA was evaluated by a UV-Vis spectrophotometer (MAPADA Instruments Co., Ltd.) at 254 nm.^5,10^

The membrane flux was calculated using eqn (2):^44^2
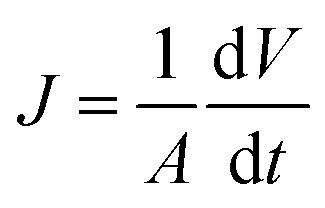
where *J* is the permeate flux [L m^−2^ h^−1^ (LMH)], *A* is the active membrane filtration area (m^2^), *V* is the total volume of permeate (m^3^), and *t* is the filtration time (min).

The rejection coefficient for HA was calculated as3
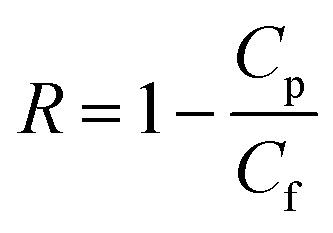
where *C*_p_ and *C*_f_ are the HA concentration in the permeate at a particular time and the initial concentration of HA in the feed, respectively.

### Photocatalytic degradation of HA

The photocatalytic degradation of HA was evaluated by determining the decrease in HA concentration in the feed solution with time. The samples from the feed were collected at regular intervals of 10 min and evaluated for the concentration of HA. The HA concentration in the feed solution was calculated using eqn (4):4
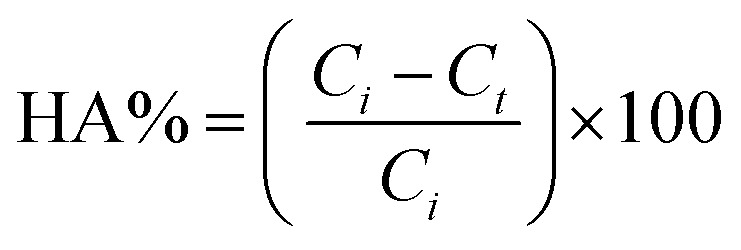
where *C*_*i*_ and *C*_*t*_ refer to the initial concentration of HA and the concentration of HA at any time *t* in the feed solution, respectively.

The photocatalytic degradation rate of HA was also studied using the first-order reaction rate as follows:5
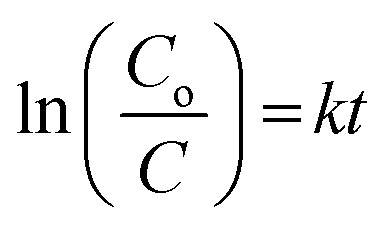


### Membrane reusability and damage analysis

To assess the long term use of the membrane, four consecutive photocatalytic UF cycles were run with the membrane. The permeate flux, HA rejection coefficient, and the corresponding concentration of HA in the feed were determined. The concentration of HA was 2 mg L^−1^ and the concentration of TiO_2_ NPs on the membrane surface was 3.04 g m^−2^. The damage analysis of the membrane was also conducted based on the filtration performance of the membrane.

## Results and discussions

### Hydrophilicity and membrane resistance

**Fig. 2:**
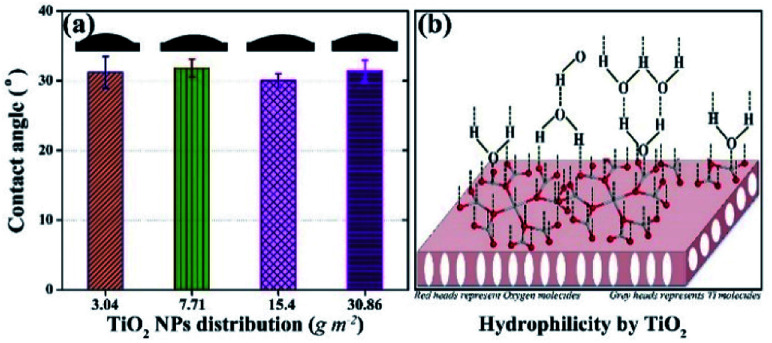
Hydrophilic potential of membranes with surface located TiO_2_ NPs: (a) contact angle values of membranes with different distributions of TiO_2_ NPs; (b) schematic of the hydrophilic mechanism by TiO_2_.

Hydrophilicity, which is assessed by water contact angle, is an important factor that influences the antifouling potential of the membrane. The contact angle was measured at five different positions on each membrane and the results are shown in Fig. 2a. The contact angles of all membranes were *ca.* 30°, whereas, the contact angles of the membrane without TiO_2_ NPs and the membrane containing 1.5 wt% TiO_2_ NPs in its matrix were reported as 66° and 57°, respectively.^43^ Scanning electron microscopy and energy dispersive X-ray spectroscopy of the membrane (3.04 g m^−2^ TiO_2_ distribution) were performed to confirm the presence of TiO_2_ NPs on surface. The results show the presence of TiO_2_ NPs on the membrane surface. The details are given in the (ESI) Fig. S1.† Thus, the presence of TiO_2_ NPs on the surface of the membranes indicated a significant increase in hydrophilicity. However, no observable differences were found in the contact angles of the membranes with different distribution of NPs. The outer surfaces of all the membranes were completely covered by TiO_2_ NPs, which induced a large hydrophilic effect in all the membranes. The hydrophilic impact by TiO_2_ is based on hydrogen bonding with adjacent water molecules, following which a thin layer of water develops on the membrane,^29,45^ as shown in Fig. 2b. The leaching and detachment of NPs from the membrane were also assessed using a filtration test with pure water for 24 h. The turbidity of the feed and permeate was calculated before starting the filtration, after 24 h, and during the filtration. The turbidity of the water sample did not increase, indicating that the NPs were neither detached nor leached from the membrane. The results are shown in ESI (Fig. S2).†

**Fig. 3:**
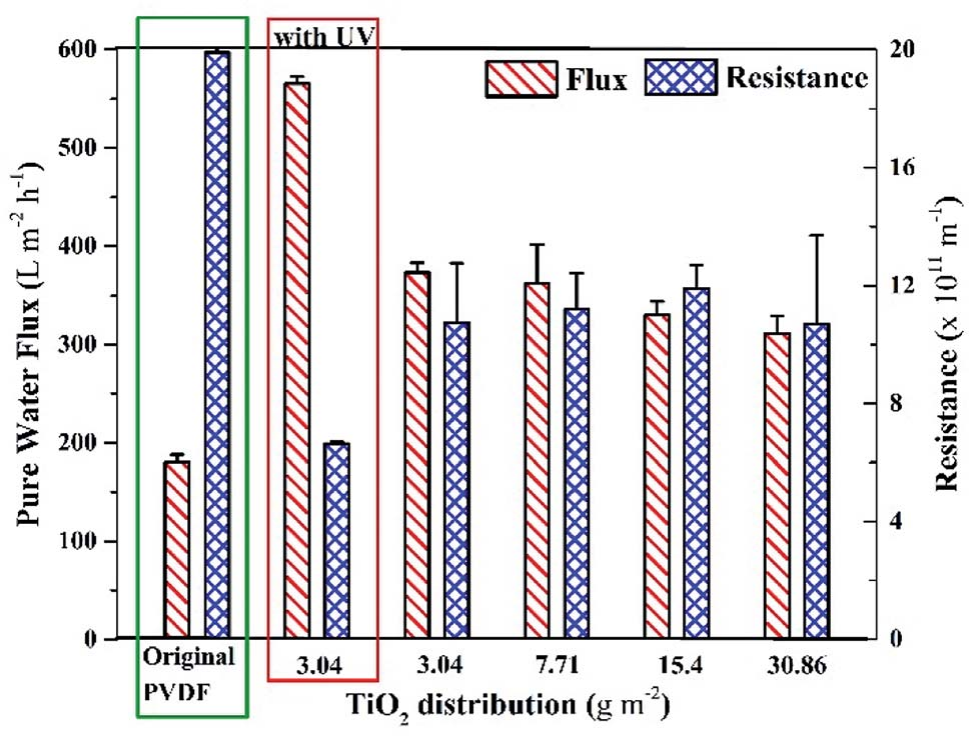
Pure water flux and internal resistances of the membranes with different distribution amount of surface-located TiO_2_ NPs in comparison with the original PVDF membrane.

The internal resistances of the membrane were estimated to assess the improvement in membrane flux. The internal resistance of the neat PVDF membrane has been reported elsewhere as 19.9 ± 0.5 × 10^11^ m^−2^,^43^ but the membranes prepared with the new method showed internal resistances of 10.6 ± 2 × 10^11^ m^−2^, 11.2 ± 1.2 × 10^11^ m^−2^, 11.9 ± 0.8 × 10^11^ m^−2^ and 10.7 ± 4 × 10^11^ m^−2^ with TiO_2_ NPs distribution on membranes as 3.04 g m^−2^, 7.71 g m^−2^, 15.4 g m^−2^, and 30.86 g m^−2^, respectively (Fig. 3). The decrease in the internal resistance of TiO_2_-containing membranes is attributed to the increase in hydrophilicity and the increase in pore size. A comparison of the pore size distribution of the membrane without TiO_2_ and the membrane with TiO_2_ is given in ESI (Fig. S3).†

The internal resistance of the membranes did not significantly change on increasing the distribution amount of TiO_2_ NPs on the membrane. Fig. 3 also shows that the pure water fluxes of all the membranes were around 350 LMH (without UV light), which were remarkably higher than that of the neat PVDF membrane (180 LMH).^43^ Moreover, it was found that the pure water flux of the membrane (with 3.04 g m^−2^ TiO_2_ distribution) was significantly higher under UV light, as shown in Fig. 3 (leftmost set of bars). The increase in the pure water flux under UV light is attributed to the effect of photoinduced hydrophilicity, where the UV light activates TiO_2_ NPs, which allows more water to pass through the membrane. In contrast, Fischer *et al.*^42^ reported around a 30% decrease in pure water flux after depositing TiO_2_ nanotubes on a PES membrane, which could be attributed to a different method of depositing nanotubes on the membrane.

The results of contact angle and membrane resistance tests demonstrated that the hydrophilicity of the membrane remained almost the same on increasing the TiO_2_ NP distribution amount from 3.04 g m^−2^. Hence, the least amount of TiO_2_ NPs distribution (*i.e.*, 3.04 g m^−2^) was considered as optimum referring to the characteristic of hydrophilicity.

### Evaluation of the photocatalytic UF process

#### Effect of TiO_2_ NP distribution on the photocatalytic UF process

**Fig. 4:**
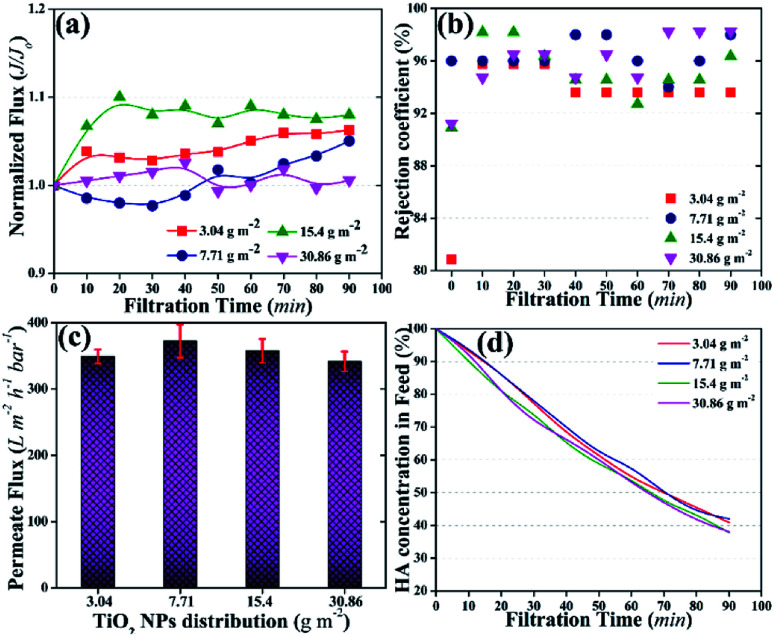
Photocatalytic UF results of the membranes with different distribution amount*s* of TiO_2_ NPs; (a) normalized fluxes of the membranes, (b) rejection coefficients of the membranes for HA, (c) permeate fluxes of the membranes at the end of the UF, (d) HA percentage in the feed tank as a factor of time, the total amount of HA in the feed was 4 mg.

The performance of the photocatalytic UF process was evaluated to observe significant variations in the results obtained from different distribution amounts of TiO_2_ NPs, and the results are shown in Fig. 4. Generally, a phenomena of flux decline starts right after actuating the filtration process,^18,24,28^ where a decline in filtration flux indicates the accumulation of pollutant molecules inside membrane pores or on the surface of the membrane, which causes an additional hindrance to solvent flow across the membrane. In this study, the membranes experienced non-conventional filtration behavior, as shown in Fig. 4a: the filtration flux started to increase instead of decreasing after turning on the photocatalytic UF process. Several undulations occurred during filtration, which indicated continuous deposition, degradation, and removal of HA on the membrane surface. However, insignificant association was observed in the results obtained with different distribution amounts of NPs on the membrane. Athanasekou *et al.*^46^ reported an increase in flux of ceramic membrane dip-coated with reduced graphene oxide-TiO_2_ NPs. It has been reported that the illumination of UV light generates highly energetic electron–hole pairs on TiO_2_ NPs that react with water molecules to produce ˙OH radicals,^45,47^ which increases the water uptake potential of the membranes;^46^ this phenomenon is known as “photoinduced hydrophilicity”. The term “photoinduced hydrophilicity” refers to an increase in water uptake potential of the membrane influenced by light.^46^ The permeate fluxes of the membranes were recorded as ∼350 LMH at the end of photocatalytic UF process (Fig. 4c), whereas the permeate fluxes were recorded as 51 and 70 LMH (under a conventional UF process) with the neat PVDF membrane and the PVDF membrane with 1.5 wt% TiO_2_ NPs inside the membrane matrix, respectively.^5,43^

The rejection coefficient for HA also increased [92–98% (Fig. 4b)] compared to that of the neat PVDF membrane (85%) and the membrane with 1.5 wt% loading of TiO_2_ NPs in the membrane matrix (90%).^43^ First, the presence of NPs on the membrane surface increased the rejection coefficient for the pollutants.^39^ Second, the activation of TiO_2_ NPs further increased the rejection of HA.^48^ The increase in rejection was attributed to the concurrent filtration and photocatalytic degradation of HA.

The concentration of HA in the feed was also detected as a factor of time. Fig. 4d shows a continuous decrease in HA concentration in the feed tank. After 90 min of photocatalytic UF, ∼40% of HA remained in the feed tank irrespective of the TiO_2_ NPs distribution amount on the membrane surface. The decrease in HA concentration in the feed could be the result of the accumulation of HA on the membrane and/or photocatalytic degradation by TiO_2_ NPs. For instance, an increasing trend in permeate flux could rule out the accumulation of HA on the membrane. Thus, photocatalytic degradation of HA might be the prime factor for the decrease in HA concentration in the feed.

#### Optimization of HA concentration as a model pollutant

**Fig. 5:**
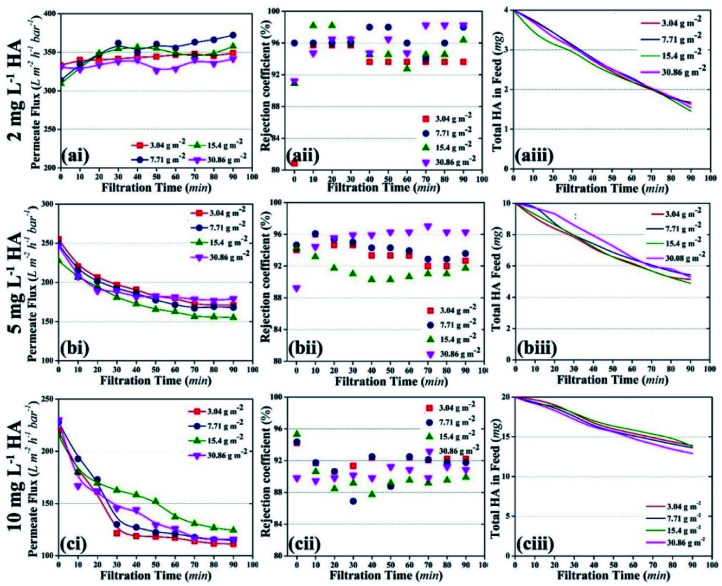
Effect of various concentrations of HA on the photocatalytic UF process; the initial ‘a’, ‘b’, and ‘c’ represent 2 mg L^−1^, 5 mg L^−1^, and 10 mg L^−1^ HA concentration, respectively; the numeric ‘i’, ‘ii’, and ‘iii’ represent permeate flux, rejection coefficient, and the decrease in HA quantity in the total feed volume, respectively.

The photocatalytic UF process was conducted with three different concentrations of HA and the results are shown in Fig. 5. It was found that the low concentration of 2 mg L^−1^ HA did not cause membrane fouling (Fig. 5ai). Correspondingly, rejection of HA was high and a total of 2.5 mg HA disappeared from the feed tank (Fig. 5aii and 5aiii). The membranes started to foul (irrespective of the TiO_2_ distribution amount) as the concentration of HA increased to 5 mg L^−1^; the rejection of HA was more than 92% while a total of 5 mg HA disappeared from the feed solution [Fig. 5bi–biii]. When the concentration of HA was increased to 10 mg L^−1^, fouling of the membrane was significantly high and a total of 6.5 mg HA disappeared from the feed [Fig. 5ci–ciii].

**Fig. 6:**
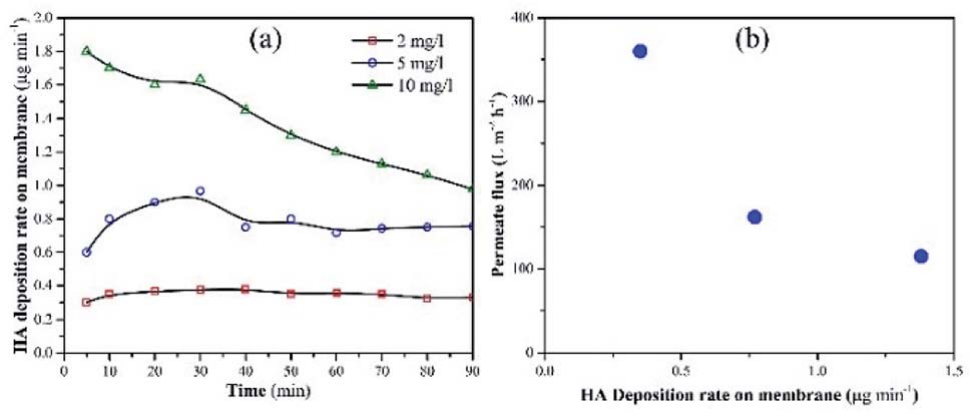
(a) Deposition rate of HA on the membrane with different concentrations of HA in the feed tank. (b) Permeate flux as a factor of HA deposition rate.

Membrane fouling was also assessed as a factor of HA deposition rate on the membrane. The deposition rate of HA on the membrane was calculated by measuring the decrease in the concentration of HA in the feed tank at a particular time. Fig. 6a shows the deposition rate of HA as a factor of time. When the concentration of HA was 2 mg L^−1^, the deposition rate of HA was stable and recorded as 0.32 μg min^−1^. When HA concentration in the feed was increased to 5 and 10 mg L^−1^, instability was found in the deposition rate. At 10 mg L^−1^ HA, the deposition rate decreased constantly. The decrease in deposition rate is attributed to the repulsion between the initially deposited HA molecules and the upcoming HA molecules. Fig. 6b shows the permeate flux relevant to the HA deposition rate.

A decrease in permeate flux was reported, which corresponds to the increase in deposition rate. Due to the higher deposition rate, HA molecules started to accumulate on the membrane and mask the NPs. As a result, UV light lost access to the NPs and failed to activate them. Finally, membrane fouling occurred and the permeate flux decreased. The increase in membrane fouling rate demonstrated that TiO_2_ NPs were unable to oxidize HA molecules when the average deposition rate of HA was 0.74 μg min^−1^ or 1.38 μg min^−1^.

#### Effect of temperature on the photocatalytic UF process

**Fig. 7:**
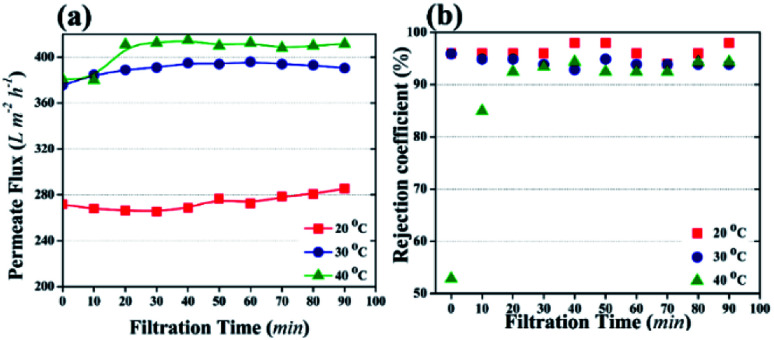
The effect of temperature on photocatalytic UF (distribution amount of TiO_2_ NPs was 3.04 g m^−2^ and HA concentration in the feed was 2 mg L^−1^). (a) Permeate flux as a factor of filtration time. (b) Rejection coefficient for HA with different filtration time.

Fig. 7 presents the results of the effect of temperature variance on the photocatalytic UF process (2 mg L^−1^ HA concentration and 3.04 g m^−2^ TiO_2_ NPs distribution amount). It was noted that the permeate flux value increased with an increase in the solution temperature (Fig. 7a) without affecting the rejection coefficient (Fig. 7b).

Temperature can affect the photocatalytic UF process by changing the viscosity of water or the oxidization of pollutants or water molecules. It is known that temperature has an inverse relationship with the viscosity of water. The kinematic viscosity of water has been reported as 1.003 mm^2^ s^−1^, 0.8 mm^2^ s^−1^, and 0.658 mm^2^ s^−1^ at 20 °C, 30 °C, and 40 °C, respectively.^49^ The decrease in viscosity allows the fast flow of water molecules across the membrane. Therefore, based on the viscosity of water, maximum flux can be attained at 40 °C. In addition, high temperature enhances the cleavage rate of water molecules by NPs and generates more ˙OH. The presence of more ˙OH increases the water uptake capacity of the membrane, resulting in high flux.

#### Photocatalytic degradation of HA

**Fig. 8:**
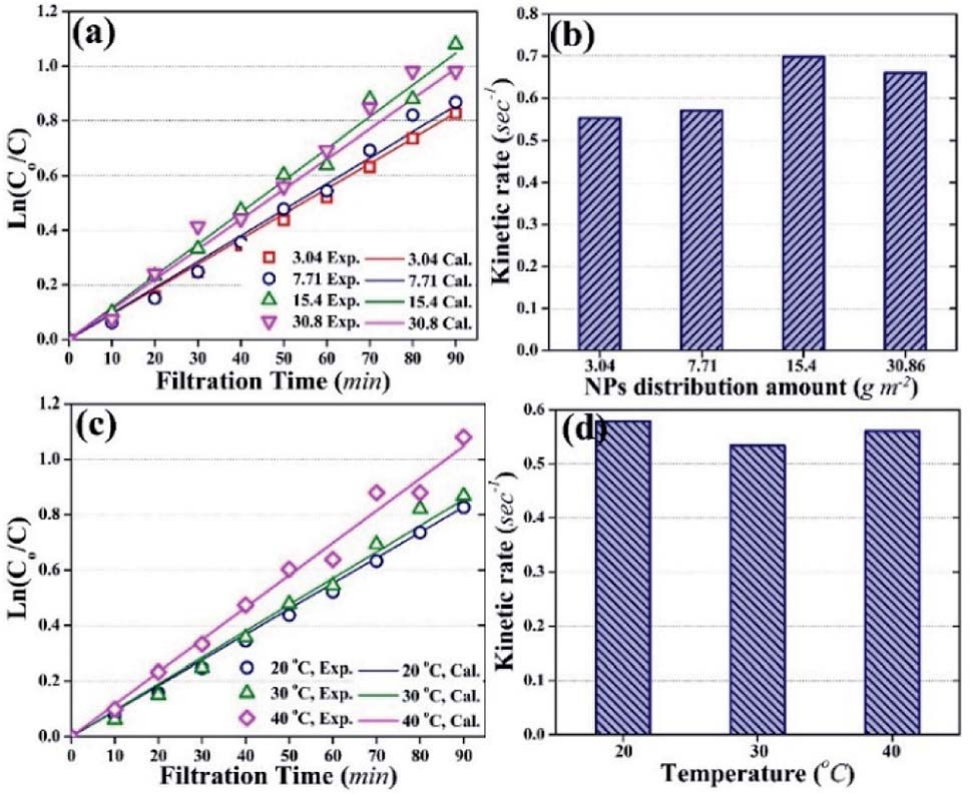
Photocatalytic degradation of HA during the photocatalytic UF process. (a) Photocatalytic degradation with different distribution amounts of TiO_2_ NPs on the membrane at room temperature (25 °C). (b) Kinetic rate of HA degradation relative to TiO_2_ NP distribution amount at room temperature (25 °C). (c) Photocatalytic degradation of HA at different temperatures. (d) Kinetic rate of HA degradation at different temperatures. Note: in (a) and (c), open symbols represent experimentally obtained values, and solid lines represent calculated values; HA concentration was 2 mg L^−1^; (c) and (d) account for the results obtained using 3.04 g m^−2^ of TiO_2_ NPs distribution amount.

Fig. 8 shows degradation rates of HA by different membranes and under different temperatures. Fig. 8a and c show graphs plotted between ln(*C*_o_/*C*) and filtration time with experimentally obtained values (open symbols) and simulated values (solid lines). Fig. 8b and d show the experimentally determined kinetic rate constants for HA degradation.

Fig. 8a demonstrates that experimentally obtained values of ln(*C*_o_/*C*) were close to the theoretically calculated values, with correlation coefficients of more than 0.1. Moreover, the photocatalytic degradation of HA followed pseudo-first order kinetics with kinetic rate constants of 0.552 s^−1^, 0.57 s^−1^, 0.69 s^−1^, and 0.66 s^−1^ for 3.04 g m^−2^, 7.71 g m^−2^, 15.4 g m^−2^, and 30.86 g m^−2^ NPs distribution amounts, respectively (Fig. 8b).

Fig. 8c shows the effect of temperature on the kinetics of photocatalytic degradation and Fig. 8d shows the kinetic rate constants for HA degradation at different temperature (membrane: 3.04 g m^−2^ NPs distribution; HA concentration: 2 mg L^−1^). The data in Fig. 8 indicate that a change in temperature did not affect the kinetics of HA degradation, and the kinetic rate constant remained almost stable at 0.55 s^−1^.

Temperature is one of the factors that can influence the photocatalytic degradation rate of pollutants.^31,50^ However, the experimental data in this study showed the insignificant effect of temperature on the photocatalytic degradation rate of HA. The results suggested that the activity of NPs remained independent of the temperature of the feed solution. To better understand the behavior of immobilized NPs at different temperatures of feed solution during the photocatalytic UF process, the Eyring-type plot can be used.

The Eyring-type plot describes the behavior of a temperature-based system in terms of its enthalpy and entropy. The graph is plotted for ln(*k*/*T*) as a factor of 1/*T*:^50^6
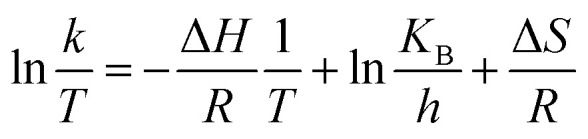
where *k* represents the pseudo-first order rate constant, *T* represents temperature, *R* is the gas law constant, *K*_B_ represents the Boltzmann constant, *h* is Plank's constant, Δ*H* is enthalpy, and Δ*S* is entropy.

**Fig. 9:**
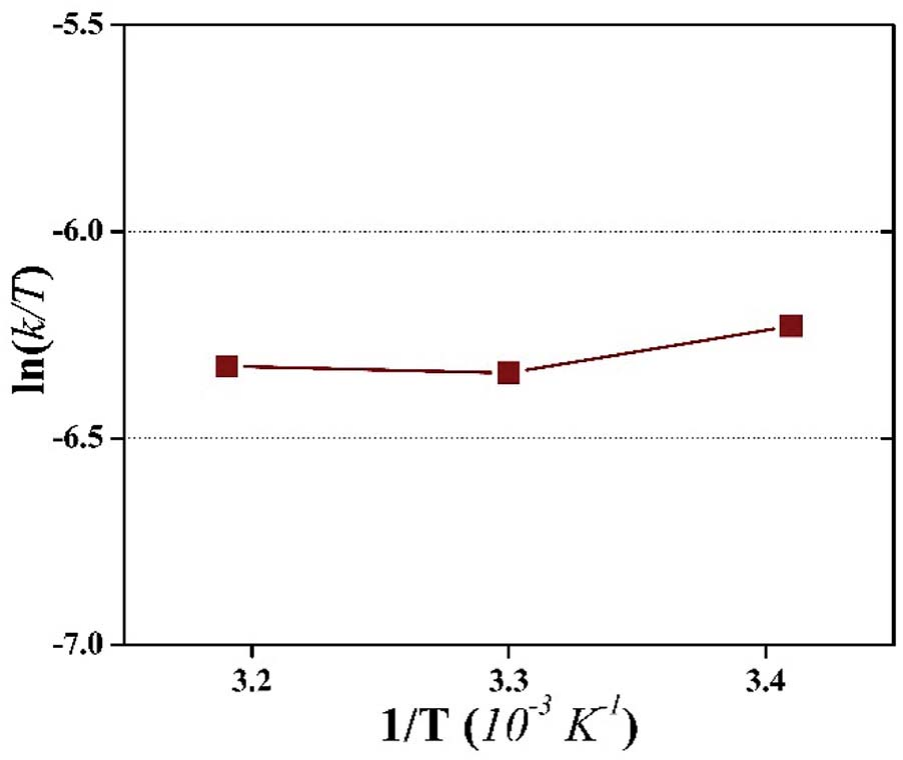
An Eyring-type plot illustrating the effect of temperature on the photocatalytic degradation of HA during the photocatalytic UF process.

The plot is used to depict the influence of temperature on photocatalytic degradation and usually gives a straight line with a negative slope.^50^ In this study, the plot showed that ln(*k*/*T*) remained constant at different temperatures, which demonstrates that the temperature did not affect the photocatalytic system (Fig. 9). The entropy, Δ*S*, and the enthalpy, Δ*H*, of the system were calculated as −145.67 J K^−1^ mol^−1^ and 0.01 kJ mol^−1^, respectively. A large negative value of Δ*S* is speculated as a reaction between the adsorbed pollutant and the surface oxidizing species photogenerated on TiO_2_ during light irradiation.^50^

On the contrary, a very small value of Δ*H* demonstrates the thermodynamic independence of the system. These observations illustrated that the activation of surface immobilized TiO_2_ remained unaffected by temperature. Therefore, it can be inferred that temperature is effective only to increase the permeate flux and not to increase the photocatalytic degradation of the pollutant. Consequently, the pollutant concentration should not exceed a particular value in order to sustain the fouling-free UF process.

#### Mechanism of photocatalytic UF

**Fig. 10:**
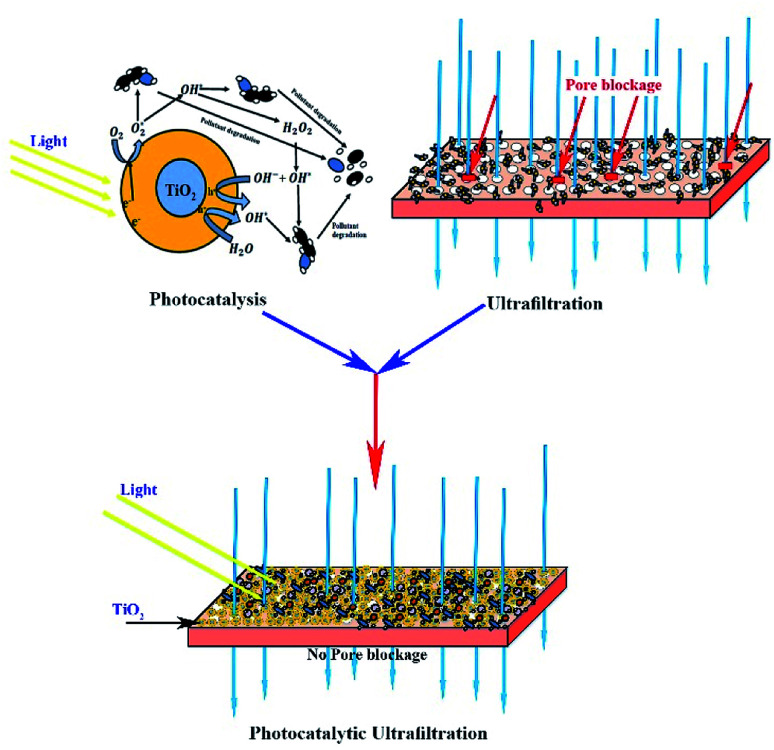
Schematic of the photocatalytic UF process.

Fig. 10 illustrates the mechanism of photocatalytic UF. It is known that the adsorption of pollutants on the membrane surface blocks the pores, resulting in a decline in flux. However, during photocatalytic UF (in this study), we found that fouling did not occur. Moreover, a large negative value of Δ*S*, calculated from the Eyring-type plot, depicted the degradation of adsorbed HA by the photogenerated surface oxidizing species. There were two phenomena working simultaneously: (i) photoinduced hydrophilicity and (ii) photocatalytic degradation. At first, the HA adsorbed on the membrane was degraded by the photocatalytic activity of NPs, which prevented membrane fouling.

Spontaneously, the hydroxyl radicals attracted more water molecules and induced more water flow through the membrane. Thus, all the results and observations imply the initial adsorption of HA on the surface of the membrane, followed by HA degradation by the photogenerated surface oxidizing species, resulting in a fouling-free UF process.

#### Membrane reusability

**Fig. 11:**
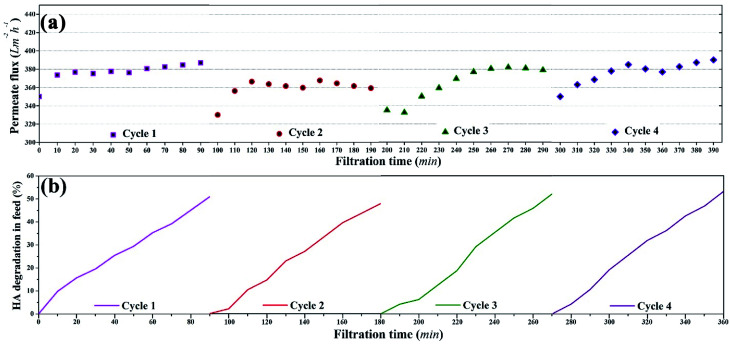
Reusability of the membrane during photocatalytic UF of HA: (a) permeate flux during four consecutive photocatalytic UF cycles, (b) HA degradation during four consecutive photocatalytic UF cycles.

The reusability of the membrane during the photocatalytic UF process was studied during four consecutive cycles and the results are illustrated in Fig. 11. In each cycle, photocatalytic UF was conducted for 90 min and after 90 min the process was stopped for 10 min. The next cycle started after the feed water was replaced with a freshly prepared feed comprising 2 mg L^−1^ HA solution. Fig. 11a shows that the permeate flux increased as a function of time and reached a constant state in four cycles, which showed the equilibrium between HA deposition and degradation on the membrane. The average flux in each cycle was recorded as 350 LMH. Fig. 11b shows the HA degradation by the membrane. It was found that *ca.* 50% HA was degraded in each cycle at a constant rate. Overall, within four consecutive cycles, it was deduced that TiO_2_ NPs continuously degraded the adsorbed HA to self-regenerate its photocatalytic potential and to continue the process of photocatalytic UF.

Overall, consistent permeate flux, stable HA rejection, and constant HA degradation were recorded during four cycles of photocatalytic UF, which confirmed the stability of the membrane for a “fouling-free UF process”. The stable and consistent performance (permeate flux and rejection) of the membrane during the four consecutive cycles also eliminated doubts regarding membrane damage.

## Conclusion

In this study, we reported the advantages of combining photocatalysis with a UF process, *i.e.*, photocatalytic UF. The photocatalytic process was induced by activation of TiO_2_ NPs immobilized on the UF membrane surface. First, the activated NPs triggered photoinduced hydrophilicity of the membrane, which allowed more solvent to pass through the membrane and increased the membrane flux during filtration. Second, the activated NPs exhibited photocatalytic degradation of adsorbed HA on the membrane surface. The continuous photocatalytic degradation of HA on the membrane surface eliminated fouling of the membrane and resulted in a fouling-free UF process. The kinetic rate constant for HA during photocatalytic UF was recorded as 0.55 s^−1^, while the HA concentration was 2 mg L^−1^. The experimental results suggest that it is necessary to maintain the HA deposition rate below 55 μg min^−1^ on membrane surface to sustain the fouling-free UF of HA. By considering the positive impact of TiO_2_ NPs activation during the UF process, the photocatalysts with high kinetic rate can be applied to further enhance the photoinduced hydrophilicity and to increase the photocatalytic degradation rate of pollutants.

## Conflicts of interest

There are no conflicts to declare.

## Supplementary Material

RA-008-C8RA03810D-s001
