# Supplementary material for: Fouling-free ultrafiltration for humic acid removal
Source: RSC Adv. 2018 Jul 11;8(44):24961–9. doi: 10.1039/c8ra03810d (PMC9082387; doi:10.1039/c8ra03810d)
Supplement: RA-008-C8RA03810D-s001 [file RA-008-C8RA03810D-s001.pdf]

Electronic Supplementary Material (ESI) for RSC Advances.
This journal is © The Royal Society of Chemistry 2018

Supporting Information

Fouling-free ultrafiltration for humic acid removal

Hassan Younas,[a,b] Jiahui Shao,[a]† Yiliang He,[a] Gul Fatima,[c] Syed Taseer Abbas Jaffar,[d] and Zohaib Ur Rehman Afridi[e]

[a] *School of Environmental Science and Engineering, Shanghai Jiao Tong University, Shanghai 200240, China*

[b]*The state key laboratory of Materials Oriented Separations, College of Chemical Engineering, Nanjing Tech University, Nanjing 210009, China*

[c]*Department of Chemistry and Chemical Engineering, Syed Ali Babar School of Science and Engineering, Lahore University of Management Sciences, Lahore 54000, Pakistan*

[d]*Department of Civil Engineering, Shanghai Jiao Tong University, Shanghai 200240, China*

[e] *Energy Management and Sustainability, U.S. Pakistan Centre For Advanced Studies in Energy, University of Engineering and Technology Peshawar, Peshawar, Pakistan*

†E. mail: jhshao@sjtu.edu.cn, Tel: +86-21-54742127

Surface characteristics of the membrane

Scanning electron microscopy (SEM) and energy dispersive X-ray spectroscopy (EDX) of the membrane was performed to make sure that TiO_2 NPs were immobilized on the surface of the membrane. The results are shown in Fig. S1. Fig S1a shows the SEM image of the membrane with 3.04 g m^{-2} TiO_2 NPs distribution on the membrane. It was found that the whole surface of the membrane was covered by TiO_2 NPs that certified the successful immobilization of TiO_2 NPs on the membrane. Fig. S1b shows the EDX mapping of the membrane surface for elemental Ti. EDX mapping also confirm that the particles in SEM image are of TiO_2 that are well spread and immobilized on the membrane surface.

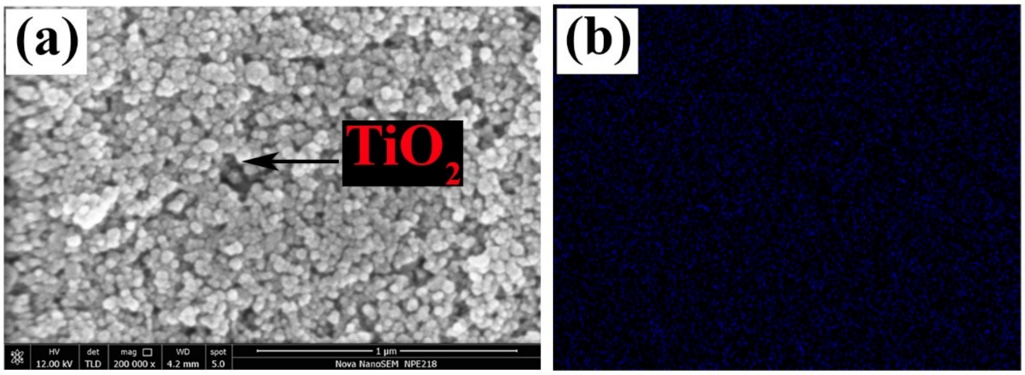

Fig. S 1. Surface of the membrane with 3.04 g m^{-2} TiO_2 distribution, (a) SEM image of the surface of the membrane, (b) EDS mapping of elemental Ti of the surface of the membrane

Stability of TiO_2 NPs attachment

Fig. S2 shows the results of stability of TiO_2 NPs attachment on the surface of the membrane. A simple yet significant test was performed to assess the attachment stability of the membrane. Cross-flow UF was performed with pure water while adjusting the pressure as 0.1 MPa and cross-flow rate as 0.5 L min^{-1}. The turbidity of the water in feed was measured before starting the filtration test and after finishing the filtration test. Whereas, turbidity of permeate was regularly measured for 24 h. The turbidity of feed was higher before starting the filtration test than the

turbidity of the feed after finishing the filtration test. The low turbidity of the feed after finishing the filtration test assured that TiO_2 NPs were firmly attached on the membrane surface and were not detached by the cross-flow pressure over the membrane. The turbidity of permeate remained lower than feed throughout the filtration test, which assured that TiO_2 NPs were not leached out of the membrane.

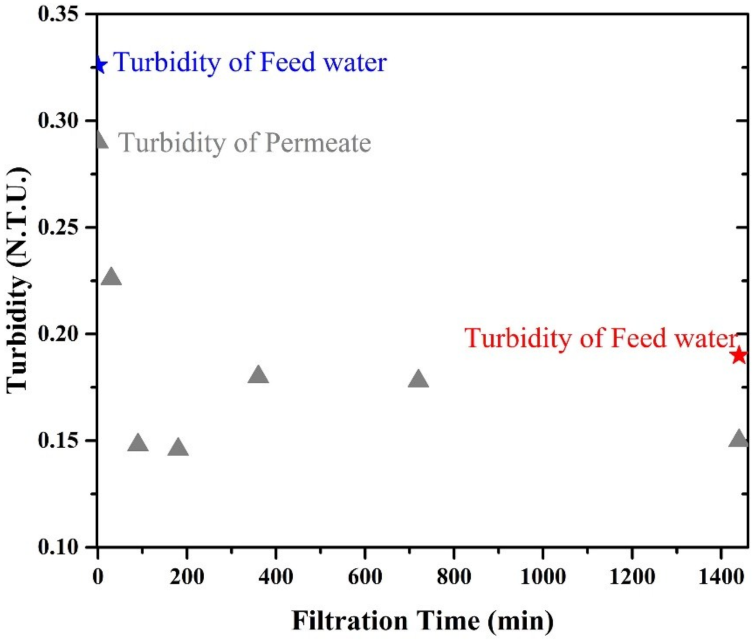

Fig. S 2. Test for the stability of TiO_2 NPs attachment on the surface of the membrane.

Pore size analysis

Fig. S3 shows the pore size distribution of the membranes. It was found that the average pore size of the membrane without TiO_2 NPs was 35 nm (Fig. S3a). Whereas, the average pore size of the membrane with TiO_2 NPs in membrane matrix and on surface of the membrane was 65 nm (Fig. S3b).

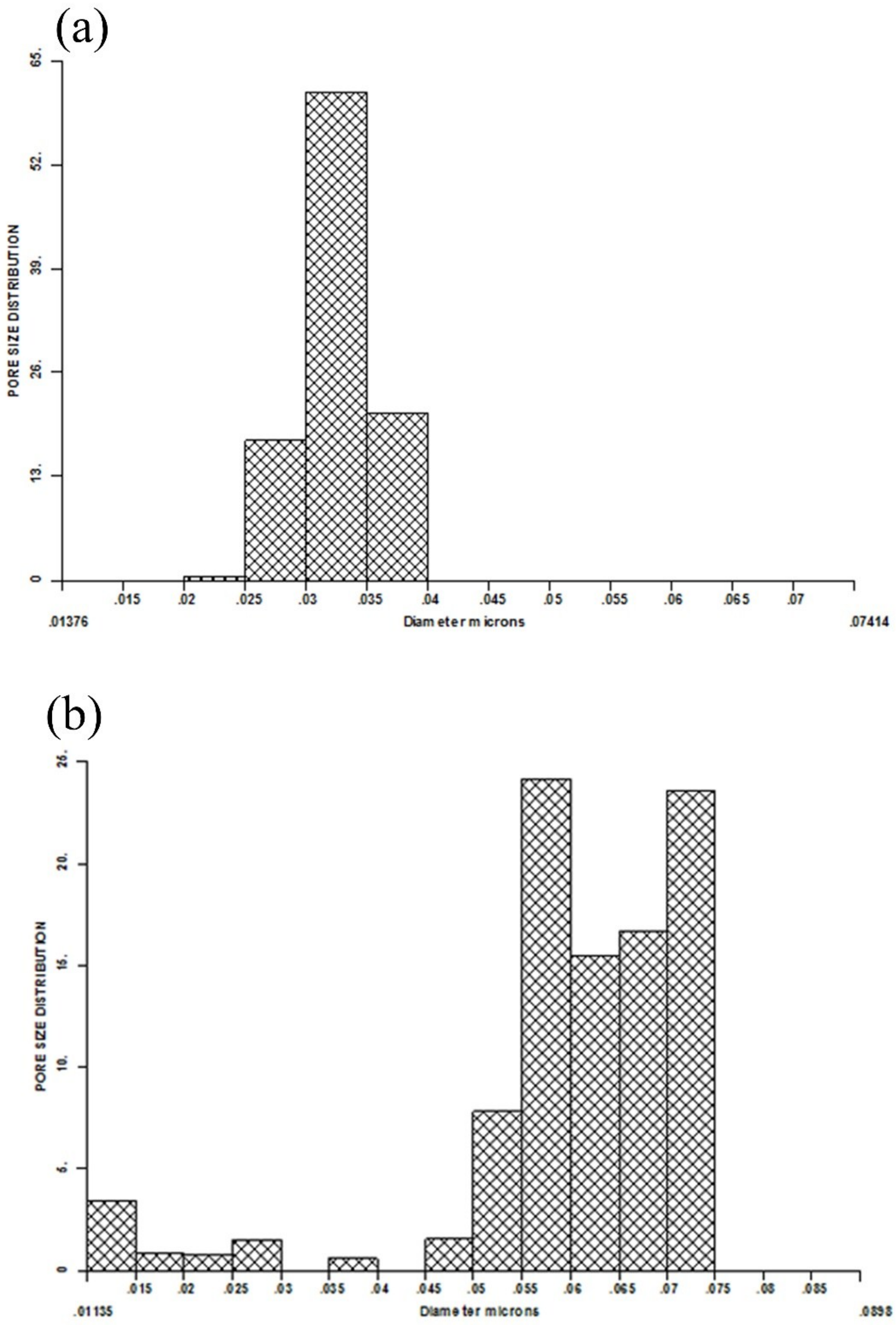

Fig. S 3. Pore size analysis of the membranes, (a) membrane without TiO_2 NPs; (b) membrane with TiO_2 NPs (TiO_2 inside membrane matrix= 1.5%, TiO_2 NPs distribution on membrane surface = 3.04 g m^{-2})
